# Trace element profile and incidence of type 2 diabetes, cardiovascular disease and colorectal cancer: results from the EPIC-Potsdam cohort study

**DOI:** 10.1007/s00394-021-02494-3

**Published:** 2021-02-15

**Authors:** Maria Cabral, Olga Kuxhaus, Fabian Eichelmann, Johannes F. Kopp, Wiebke Alker, Julian Hackler, Anna P. Kipp, Tanja Schwerdtle, Hajo Haase, Lutz Schomburg, Matthias B. Schulze

**Affiliations:** 1 Department of Molecular Epidemiology, German Institute of Human Nutrition Potsdam Rehbruecke, 14558 Nuthetal, Germany; 2 TraceAge-DFG Research Unit on Interactions of Essential Trace Elements in Healthy and Diseased Elderly, Potsdam-Berlin-Jena, Germany; 3 German Center for Diabetes Research (DZD), München-Neuherberg, Germany; 4 Department of Food Chemistry, Institute of Nutritional Science, University of Potsdam, 14558 Nuthetal, Germany; 5 Department of Food Chemistry and Toxicology, Institute of Food Technology and Food Chemistry, Technische Universität Berlin, 13355 Berlin, Germany; 6 Institute for Experimental Endocrinology, Charité-Universitätsmedizin Berlin, corporate member of Freie Universität Berlin, Humboldt-Universität Zu Berlin, and Berlin Institute of Health, CVK, Augustenburger Platz 1, 13353 Berlin, Germany; 7 Department of Molecular Nutritional Physiology, Institute of Nutritional Sciences, Friedrich Schiller University Jena, 07743 Jena, Germany; 8 Institute of Nutritional Science, University of Potsdam, 14558 Nuthetal, Germany

**Keywords:** Trace elements, Prospective study, Type 2 diabetes mellitus, Cardiovascular disease, Colorectal cancer

## Abstract

**Purpose:**

We aimed to examine the prospective association between manganese, iron, copper, zinc, iodine, selenium, selenoprotein P, free zinc, and their interplay, with incident type 2 diabetes (T2D), cardiovascular disease (CVD) and colorectal cancer (CRC).

**Methods:**

Serum trace element (TE) concentrations were measured in a case-cohort study embedded within the EPIC-Potsdam cohort, consisting of a random sub-cohort (*n* = 2500) and incident cases of T2D (*n* = 705), CVD (*n* = 414), and CRC (*n* = 219). TE patterns were investigated using principal component analysis. Cox proportional hazard models were fitted to examine the association between TEs with T2D, CVD and CRC incidence.

**Results:**

Higher manganese, zinc, iodine and selenium were associated with an increased risk of developing T2D (HR Q5 vs Q1: 1.56, 1.09–2.22; HR per SD, 95% CI 1.18, 1.05–1.33; 1.09, 1.01–1.17; 1.19, 1.06–1.34, respectively). Regarding CVD, manganese, copper and copper-to-zinc ratio were associated with an increased risk (HR per SD, 95% CI 1.13, 1.00–1.29; 1.22, 1.02–1.44; 1.18, 1.02–1.37, respectively). The opposite was observed for higher selenium-to-copper ratio (HR Q5 vs Q1, 95% CI 0.60, 0.39–0.93). Higher copper and zinc were associated with increasing risk of developing CRC (HR per SD, 95% CI 1.29, 1.05–1.59 and 1.14, 1.00–1.30, respectively). Selenium, selenoprotein P and selenium-to-copper-ratio were associated to decreased risk (HR per SD, 95% CI 0.82, 0.69–0.98; 0.81, 0.72–0.93; 0.77, 0.65–0.92, respectively). Two TE patterns were identified: manganese–iron–zinc and copper–iodine–selenium.

**Conclusion:**

Different TEs were associated with the risk of developing T2D, CVD and CRC. The contrasting associations found for selenium with T2D and CRC point towards differential disease-related pathways.

**Supplementary Information:**

The online version contains supplementary material available at 10.1007/s00394-021-02494-3.

## Introduction

Trace elements (TEs) exert a variety of cellular key functions, although they account for a very small fraction of the total body weight. TEs mediate vital biochemical reactions by acting as cofactors or catalyst for many enzymes and act as centers for stabilizing structures of enzymes and proteins. Consequently, imbalance in TE metabolism and homeostasis (deficiency or excess) may play an important role in a variety of diseases [1].

Although previous literature has suggested a link between different TEs and major age-related chronic diseases, such as type 2 diabetes (T2D), cardiovascular disease (CVD) and cancer, there appears to be substantial heterogeneity of findings from observational studies and trials. Selenium (Se) intake, for example, has been postulated to be protective for cancer development. Overall, case–control and prospective cohort studies suggest an inverse association of Se with cancer risk [2, 3], but randomized controlled trials (RCTs) do not fully support this notion. Meta-analyzing all RCTs on Se supplementation and cancer, intervention groups had no statistically different risk than control groups [4]. In case of manganese (Mn), supplementation has been shown to increase insulin secretion to improve glucose tolerance under conditions of dietary stress in animal models [5]. In humans, a recent case–control study suggested a U-shaped association of plasma Mn levels and diabetes with both low and high levels indicating higher diabetes risk [6].

The discrepancy between observational studies and intervention trials may be explainable by confounding bias in the former. Also, trial results may have been inconclusive due to low power to observe moderate effects and the inclusion of individuals with sufficient TE status. More strikingly, neither prospective observational studies nor randomized trials seem to have considered several TEs at the same time.

Attention has been recently directed to the importance of considering co-exposure of several toxicants in epidemiological studies [7–9], as failure to capture potential interactive effects of exposure may prevent the understanding of the etiopathogenic mechanisms related to many nutritional disorders [10]. However, the interplay between TEs has been sparsely characterized, thereby beneficial or detrimental effects in observational studies may also reflect effects of other correlated TEs or their interaction. For example, serum concentrations of copper (Cu) are strictly regulated by compensatory mechanisms that insure its concentration within certain ranges of nutritional intake. This changes under inflammatory conditions where specific mechanisms decrease serum concentration of zinc (Zn) and increase serum concentration of Cu [11]. Hence, a common feature of several age-related chronic diseases appears to be an increase of the Cu-to-Zn ratio [11].

Based on these considerations, we aimed to investigate the prospective association of selected TEs [Mn, iron (Fe), Cu, Zn, iodine (I), Se] and functional TE markers [Selenoprotein P (SELENOP) and Free Zinc (Free Zn)], as well as their interdependence, with incident T2D, CVD [myocardial infarction (MI) and stroke] and colorectal cancer (CRC) using data from the European Prospective Investigation into Cancer and Nutrition (EPIC)-Potsdam cohort.

## Methods

### Study design and population

The EPIC-Potsdam cohort is part of the multicenter EPIC study [12]. In Potsdam and the surrounding area, 27,548 persons were recruited from the general population (16,644 women and 10,904 men) between 1994 and 1998, with an age range of 35–64 years [13]. The baseline examination included anthropometric measurements, a validated semi-quantitative food frequency questionnaire (FFQ) [14], a lifestyle questionnaire, and a personal interview. Written informed consent was obtained from all study participants a priori, and the study was approved by the ethics committee of the Medical Society of the State of Brandenburg [13]. In course of active follow-up, participants were contacted every 2 years, with response rates ranging between 90 and 96% per follow-up round [15]. The association between TE profile and incident chronic disease was evaluated using a case-cohort design [16] with blood samples from a random sub-cohort of EPIC-Potsdam participants (*n* = 2500) and incident cases of T2D, CVD and CRC. Individuals were excluded from the analyses if they had insufficient or no serum; unclear disease status; prevalent T2D, cancer, MI, or stroke at baseline, or incomplete follow-up information (Supplementary Fig. 1). The analytic samples comprised 2741 participants for T2D (2090 sub-cohort participants and 705 incident T2D cases, overlap: 54), 2464 participants for CVD (2087 sub-cohort participants and 414 incident CVD cases, overlap: 37), and 2309 participants for CRC (2106 sub-cohort participants and 219 incident CRC cases, overlap: 16). Follow-up extended until August 2005 for T2D (median, interquartile range: of 6.6, 2.7 years); November 2006 for CVD (8.3, 1.6 years), and December 2009 for CRC (10.7, 1.6 years).

### Blood collection and laboratory analysis

At baseline, trained study personnel obtained 30 mL peripheral venous blood from each participant. Blood was separated into serum, plasma (with 10% of total volume citrate) and blood cells and was subsequently stored in tanks of liquid nitrogen at − 196 °C or in deep freezers at − 80 °C until time of analysis. For TE-profiling, the method published by Kopp et al. [17] was employed [18]. In brief, 50 µL of serum sample was diluted with 440 µL of a diluent solution as described in [17]. As internal standard and for isotope dilution analysis 10 µL of a solution containing 50 µg/L ^77^Se and 5 µg/L Rh was added to give a total volume of 500 µL. This solution was directly subjected to analysis via inductively coupled plasma tandem mass spectrometry (ICP-MS/MS) (Agilent ICP-QQQ-MS 8800, Agilent Technologies, Waldbronn, Germany). For external calibration (all elements except Se), standards were prepared matrix-matched in the diluent solution. Se was determined using isotope dilution analysis. For quality control, reference material RECIPE^®^ ClinChek^®^ serum control lyophilized (Ref. 8880-8882, Lot 347 or Lot 1497, each in both levels) was measured in triplicate daily. Mean recoveries were Mn: 98.5 ± 11.1%, Fe: 100.2 ± 7.0%, Cu: 95.5 ± 6.8%, Zn: 96.9± 6.1%, I: 105.9 ± 16.4%, Se: 97.3 ± 7.9%. Furthermore, sufficient blank samples (distilled H2O) were carried along to determine limits of detection (LOD, 3ϭ-criterion) and quantification (LOQ, 10ϭ-criterion) on a daily basis. SELENOP concentrations were measured using a validated sandwich ELISA (selenOtest ELISA, selenOmed GmbH, Berlin, Germany) characterized recently in detail [19], which was independently proven as a highly reliable commercial assay [20]. Free Zn concentration was determined by the low-molecular-weight fluorescent probe Zinypr-1 as reported before [21], with the following modifications: The incubation times for *F*, *F*_min_ and *F*_max_ were set to 30, 20 and 30 min, respectively. For the induction of *F*_min_ 15 µL EDTA-solution (stock 800 µM, final concentration 104 µM) and for *F*_max_ 15 µL ZnSO_4_-solution (stock 4.5 mM; final concentration 0.52 mM) were added per well. Plasma concentrations of high-density lipoprotein cholesterol (HDL-C) and high-sensitivity CRP (hsCRP), as well as the percentage of glycated hemoglobin (HbA1c) were measured at the Department of Internal Medicine, University of Tübingen (Tübingen, Germany) with an automatic ADVIA 1650 analyzer (Siemens Medical Solutions, Erlangen, Germany) in 2007 [22]. All biomarker measurements conducted in plasma were corrected for the dilution introduced by citrate volume to improve comparability with concentrations measured in EDTA–plasma reported in the literature [23]. Because some incident CRC cases had these biomarkers measured at a later stage, potential differences in the old and new measurements were evaluated in a subsample of 31 cases and 30 controls with repeated measurements. The values were corrected using the median of the differences in old and new values (− 0.12% for HbA1c, − 0.023 mg/L for hsCRP and 5.74 mg/dL for HDL-C, respectively). Laboratory measurements were conducted by experienced technical personnel following the manufacturer’s instructions.

### Outcome ascertainment

Incident cases of diabetes were identified during follow-up via self-reports of a diabetes diagnosis, diabetes-relevant medication, or dietary treatment due to diabetes. All incident cases were verified by questionnaires mailed to the diagnosing physician asking about the date and type of diagnosis, diagnostic tests, and treatment of diabetes. Only cases with a physician diagnosis of T2D, classified according to the *International Statistical Classification of Diseases, 10th Revision* (ICD10: E11) and a diagnosis date after the baseline examination were considered as confirmed incident cases of T2D. Incident MI and stroke cases were identified by self-report in follow-up questionnaires or by death certificate. To increase sensitivity, the questionnaire included additional questions about typical stroke symptoms [12, 24]. All self-reports for CVD cases were verified by contacting the patients’ treating physicians or by review of death certificates according to *World Health Organization Monitoring of Trends and Determinants in Cardiovascular Disease criteria* [25]. Cases were classified as incident MI (ICD-10 I21), ischemic stroke (IS) (ICD-10 I63.0 to I63.9), hemorrhagic stroke (ICD-10 I60.0 to I61.9), or undetermined stroke (ICD-10 I64.0 to I64.9) by two physicians in the study center [26]. Only confirmed cases were considered for analysis. CVD cases were calculated by combining the verified incident cases of MI and stroke depending on whichever occurred first. Accordingly, to verify cancer status, once a participant was identified as a potential case, a standard inquiry form was sent to the treating physician and then evaluated by study physicians. CRC incident cases comprised diagnoses of carcinomas of the proximal colon (codes C18.0–18.5), distal colon (codes C18.6 and C18.7), and rectum (codes C19 and C20). Follow-up was defined as the time between enrollment and study exit, which was determined by diagnosis of the respective disease, death, dropout, or the censoring date, whichever occurred first.

### Baseline anthropometry and lifestyle characteristics

Baseline measurement of anthropometric parameters was obtained by trained personnel with the participants dressed in light clothes and barefoot. BMI was calculated as the ratio of body weight (kg) to height squared (m^2^). Waist circumference was measured midway between the lower rib margin and the superior anterior iliac spine to the nearest 0.5 cm with a non-stretching tape applied horizontally.

Measurements of systolic and diastolic blood pressure (BP) were obtained after a resting period of 15–30 min [27]. History of hypertension was defined as systolic BP ≥ 140 mmHg, diastolic BP ≥ 90 mmHg, self-reported hypertension diagnosis, or use of antihypertensive medication.

Information on educational attainment, smoking, medication and leisure time physical activity was assessed with a self-administered questionnaire and a personal interview by trained interviewers using a computer-assisted interview [12]. We considered sport activities, cycling and gardening as leisure time activities, calculated as the average time spent per week during the 12 months before the baseline recruitment.

A food frequency questionnaire (FFQ) measuring usual diet over the past 12 months was administered to the participants to collect information on amount and frequency of food and beverage intake, at baseline [14, 28]. Information regarding supplement use was also collected from this FFQ. In particular, participants were asked whether they were regularly (continuously for at least 4 weeks) taking the following preparations: mineral tablets (yes/no), vitamin tablets (yes/no). A diet score (referring to the Mediterranean diet score adapted to non-Mediterranean populations) was computed at baseline to study overall healthy diet as a determinant of TE status. Construction of this score has been fully described elsewhere [29].

### Statistical analysis

#### Descriptive statistics

General demographic and laboratory characteristics were summarized as mean ± standard deviation (SD) or as median with interquartile range (IQR), depending on the normality of the continuous variables. Categorical variables were summarized as proportions.

#### Treatment of left-censored and missing data

For Mn, 483 concentration values were below the LOD, and 364 concentration values were below the LOQ. Left-censored data were handled by substituting by LOD/√2 for censored values less than LOD and by LOQ/√2 for censored values less than LOQ. Missing values (⁓ 10% of the total sample) were handled using multiple imputation based on the fully conditional specification method [30]. SAS PROC MIANALYZE^®^ was used to combine the results of the analyses on 5 imputed datasets and to generate valid statistical inferences.

#### Assessment of TE markers interdependence

The relationship between TE markers was investigated through age and sex-adjusted Spearman’s correlation coefficients in the full sample (*n* = 3834), followed by principal component analysis (PCA). Briefly, PCA is a major dimension reduction technique that aggregates variables on the basis of the degree to which they are correlated with one another. The goal is to identify linear composites of optimally weighted variables (principal components) that account for the largest amount of variation in TEs between participants. The factors were orthogonally rotated with the ‘VARIMAX option’, using the PROC FACTOR^®^ procedure in SAS. The number of factors retained was based on eigenvalue > 1, a scree-test and the interpretability of factors.

#### TE profile and risk of type 2 diabetes, cardiovascular disease and colorectal cancer

We used Cox proportional hazards regression models to estimate multivariable-adjusted hazard ratios (HRs) and 95% confidence intervals (CIs) for the associations of TEs with each incident chronic disease (T2D, CVD and CRC). Possible nonlinear relationships were further examined with restricted cubic splines, with three knots fitted at the 10th, 50th and 90th percentile of TE distribution, and the cubic spline and linear models were compared using likelihood ratio test. The associations were estimated modelling individual TEs as well as identified TE patterns continuously for which a logarithmic transformation and a Z-standardization (mean = 0, SD = 1) were used to improve normality and comparability, and categorically (according to quintiles of TE distribution in the subcohort) for the associations that deviated from linearity. To account for the case-cohort study design, weights were assigned using the approach proposed by Prentice [16, 31], and robust variance estimators were used to calculate 95% CIs using the methods described by Lin and Wei [32]. We assessed Schoenfeld residuals to validate the appropriateness of the proportional hazard´s assumption. We defined the dependent time variable as the time period between age of recruitment and the age of exit (age of diagnoses or age of death or censoring). To be less sensitive to violations of the HR, the models were stratified by age in years. Sex, education (no degree/vocational training, trade/technical school, university degree), BMI, waist circumference, smoking status (never smokers, ex-smokers, current smokers), overall leisure-time physical activity (defined as the sum of sports, biking and gardening in h/week), alcohol consumption categories according to recommended upper limits (0 g, < = 12 g for women/< = 24 g for men, > 12 g for women/> 24 g for men), prevalent hypertension (yes or no), anti-hypertensive and lipid-lowering medication (yes or no), vitamin and mineral preparations (yes or no), and dietary quality (assessed by the Mediterranean score) were considered as covariates in the Cox models to account for potential confounding. By testing cross-product terms in the fully adjusted model, we did not detect evidence for effect modification by age or sex and therefore present results from pooled models.

In addition, we assessed the relationship of TEs with HbA1c, HDL-C and hsCRP through age and sex-adjusted spearman correlations, to explore possible mediating effects of metabolic markers.

In a sensitivity analysis, we repeated all the analyses excluding TEs higher than the 99th percentile.

All statistical analyses were performed using the statistical software package SAS (version 9.4, Enterprise Guide 7.1, SAS Institute Inc., Cary, NC, USA), with a significance level of 0.05 for 2-sided tests.

## Results

### Characteristics of the participants

Baseline characteristics of the subcohort according to median concentrations of TEs are presented in Table 1. No major differences were observed regarding TE concentrations and the observed characteristics, with the exception of Cu. Participants with higher Cu concentrations were more likely to be women, have lower educational attainment and lower alcohol consumption.

**Table 1:** Baseline characteristics of the EPIC-Potsdam subcohort according to median concentrations of trace elements, imputed sample, *n* = 2087

	Manganese (µg/L)		Iron (µg/L)		Copper (µg/L)		Zinc (µg/L)	
	Median (IQR): 1.04 (1.14)		Median (IQR): 928 (433)		Median (IQR): 1021 (333)		Median (IQR): 728 (185)	
	< 1.04	≥ 1.04	< 928	≥ 928	< 1021	≥ 1021	< 728	≥ 728
Female (%)	65.2	59.8	67.2	57.9	42.1	82.9	66.4	58.7
Age (years)^a^	50.5 (15.5)	47.9 (15.6)	48.8 (14.4)	49.0 (16.4)	49.0 (15.0)	48.7 (16.1)	48.5 (15.2)	49.8 (15.8)
BMI (kg/m^2^)^b^	25.9 (4.3)	25.9 (3.9)	25.9 (4.3)	25.8 (3.8)	25.7 (3.8)	26.1 (4.4)	25.7 (4.2)	26.0 (4.0)
Waist circumference (cm)^b^	84.6 (12.5)	85.6 (12.5)	84.7 (12.8)	85.5 (12.3)	87.0 (12.6)	83.2 (12.1)	84.3 (12.6)	85.9 (12.4)
University degree (%)	38.5	40.6	38.0	41.0	46.0	33.1	39.3	39.8
Leisure-time physical activity (h/week)^a^	4.5 (6.0)	4.5 (6.5)	4.5 (6.0)	5.0 (6.5)	5.0 (6.5)	4.0 (6.0)	4.5 (6.0)	5.0 (6.5)
Use of vitamin supplement, yes (%)	17.8	15.3	16.8	16.4	17.5	15.7	15.9	17.3
Use of mineral preparation, yes (%)	12.6	11.3	12.1	11.8	10.8	13.1	10.1	13.7
Current smokers, %	20.0	20.6	20.1	20.6	21.0	19.6	19.1	21.5
High alcohol consumption (> 12 g for women, > 24 g for men) (%)	26.5	28.5	25.6	29.4	31.3	23.6	26.1	28.8
Prevalent hypertension (%)	48.9	42.9	43.4	48.4	45.1	46.8	43.6	48.2
Anti-hypertensive medication (%)	18.9	13.6	16.0	16.5	14.5	18.0	15.7	16.8
Lipid-lowering medication (%)	4.1	3.4	3.3	4.2	4.0	3.4	3.8	3.7
Mediterranean score^b^	9.0 (2.7)	8.9 (2.7)	8.9 (2.8)	9.0 (2.7)	9.1 (2.8)	8.8 (2.7)	9.0 (2.7)	9.0 (2.7)

	Iodine (µg/L)		Selenium (µg/L)		Selenoprotein P (mg/L)		Free zinc (nM)	
	Median (IQR): 56.7 (15.9)		Median (IQR): 80.0 (19.1)		Median (IQR): 5.3 (1.8)		Median (IQR): 0.59 (0.31)	
	< 56.7	≥ 56.7	< 80.0	≥ 80.0	< 5.3	≥ 5.3	< 0.59	≥ 0.59
Female (%)	51.5	73.5	61.7	63.4	67.4	57.3	60.3	64.9
Age (years)^a^	50.4 (15.0)	47.9 (15.7)	49.4 (16.2)	48.5 (14.9)	47.8 (15.5)	50.7 (15.0)	48.9 (16.0)	48.8 (15.0)
BMI (kg/m^2^)^b^	26.0 (4.1)	25.8 (4.1)	25.9 (4.3)	25.9 (3.9)	25.6 (4.2)	26.2 (4.0)	26.1 (4.3)	25.6 (3.9)
Waist circumference (cm)^b^	86.6 (12.7)	83.6 (12.2)	85.2 (12.9)	85.0 (12.1)	83.7 (12.6)	86.6 (12.3)	86.1 (13.0)	84.1 (12.0)
University degree (%)	42.7	39.4	38.7	40.4	37.9	41.3	38.8	40.3
Leisure-time physical activity (h/week)^a^	5.0 (6.5)	4.0 (5.5)	5.0 (6.5)	4.5 (6.0)	4.5 (6.0)	5.0 (6.5)	4.5 (6.0)	5.0 (6.3)
Use of vitamin supplement, yes (%)	16.4	16.7	14.9	18.2	15.2	18.1	15.4	17.8
Use of mineral preparation, yes (%)	11.4	12.5	11.3	12.6	10.8	13.1	10.2	13.7
Current smokers, %	20.5	20.2	20.4	20.2	20.2	20.4	20.3	20.3
High alcohol consumption (> 12 g for women, > 24 g for men) (%)	32.9	22.1	27.1	27.9	27.1	27.9	29.1	25.8
Prevalent hypertension (%)	47.2	44.6	44.1	47.7	41.8	50.3	47.1	44.7
Anti-hypertensive medication (%)	16.4	16.1	16.4	16.1	13.4	19.3	16.6	15.9
Lipid-lowering medication (%)	4.0	3.4	3.0	4.5	3.1	4.5	3.4	4.1
Mediterranean score^b^	9.1 (2.7)	8.9 (2.7)	8.8 (2.7)	9.2 (2.7)	8.9 (2.7)	9.1 (2.7)	9.0 (2.7)	9.0 (2.8)

Subcohort number for CVD outcome. Different number in subcohort participants due to outcome specific exclusions. Data are expressed as medians (IQR)^a^ or means (SD)^**b**^ for continuous variables and % for categorical variables

*BMI* body mass index

### Interdependence of serum TE and other biomarkers

Age and sex-adjusted Spearman’s correlation coefficients between TEs and potential mediators are shown in Table 2.

**Table 2:** Age and sex-adjusted Spearman correlations between TE and metabolic biomarker concentrations, EPIC-Potsdam, imputed sample, *n* = 3834

	Mn	Fe	Cu	Zn	I	Se	SELENOP	Free-Zn	HbA1c	HDL-C	HsCRP
Mn	1										
Fe	0.16	1									
Cu	0.06	0.06	1								
Zn	0.04	0.25	0.12	1							
I	0.04	0.10	0.43	0.17	1						
Se	− 0.06	0.08	0.20	0.26	0.27	1					
SELENOP	− 0.01	0.01	0.06	0.11	0.06	0.45	1				
Free-Zn	− 0.01	0.06	− 0.03	0.33	0.01	0.03	− 0.03	1			
HbA1c	0.11	− 0.06	0.07	0.05	0.02	0.03	0.14	− 0.01	1		
HDL-C	0.02	0.03	0.03	− 0.04	− 0.04	0.00	− 0.04	0.03	− 0.16	1	
HsCRP	0.07	− 0.11	0.43	− 0.03	0.18	0.01	0.03	− 0.07	0.21	− 0.13	1

*Mn* manganese, *Fe* iron, *Cu* copper, *Zn* zinc, *I* iodine, *Se* selenium, *SELENOP* selenoprotein P, *Free Zn* free zinc, *HDL-C* high-density lipoprotein cholesterol, *HbA1c* glycated hemoglobin, *HsCRP* high-sensitivity C-reactive protein

Correlations between TEs and functional biomarkers (SELENOP and Free-Zn) were low to modest and ranged from − 0.06 to 0.45. The strongest correlation was observed between Se and SELENOP (*r* = 0.45), followed by Cu and I (*r* = 0.43) and Zn and Free-Zn (0.33). Cu and hsCRP were moderately correlated (*r* = 0.43).

**Fig. 1:**
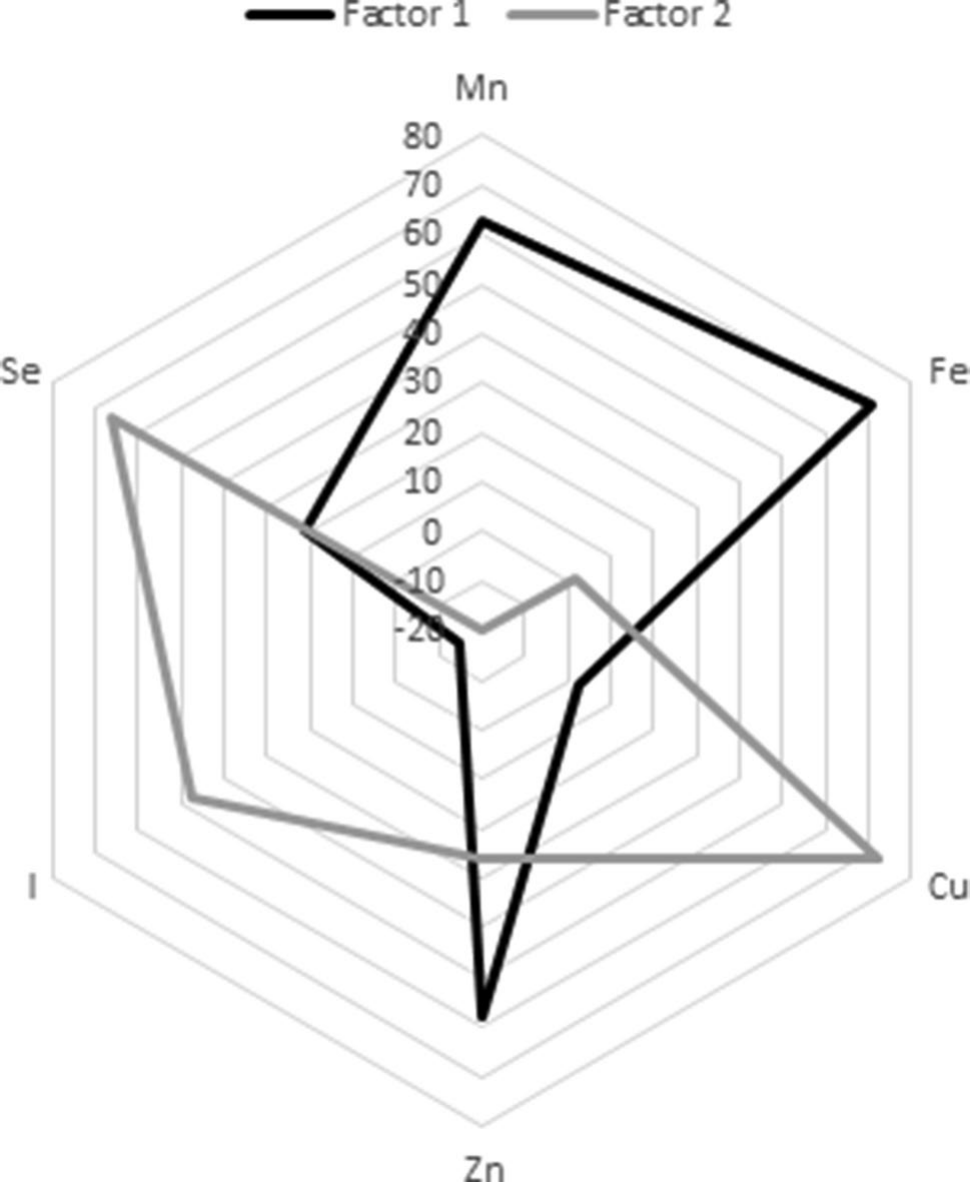
Factor loadings after varimax rotation of the two PCA-derived factors based on TE concentrations at baseline, EPIC-Potsdam, imputed sample, *n* = 3834. *Mn* manganese, *Fe* iron, *Cu* copper, *Zn* zinc, *I* iodine, *Se* selenium

We identified two patterns of TEs by PCA that accounted for 43% of the total variance. The first pattern was mainly related to higher concentrations of Mn, Fe and Zn and the second pattern to higher concentrations of Cu, I and Se (Fig. 1).

### TEs and risk of type 2 diabetes

In the spline regression analysis, the shape of the observed associations of Fe, Cu, Zn, Se, SELENOP, Free Zn, Cu-to-Zn ratio and Se-to-Cu ratio with T2D was linear, while associations of Mn, and factor 2 deviated from linearity (Supplementary Fig. 2).

**Fig. 2:**
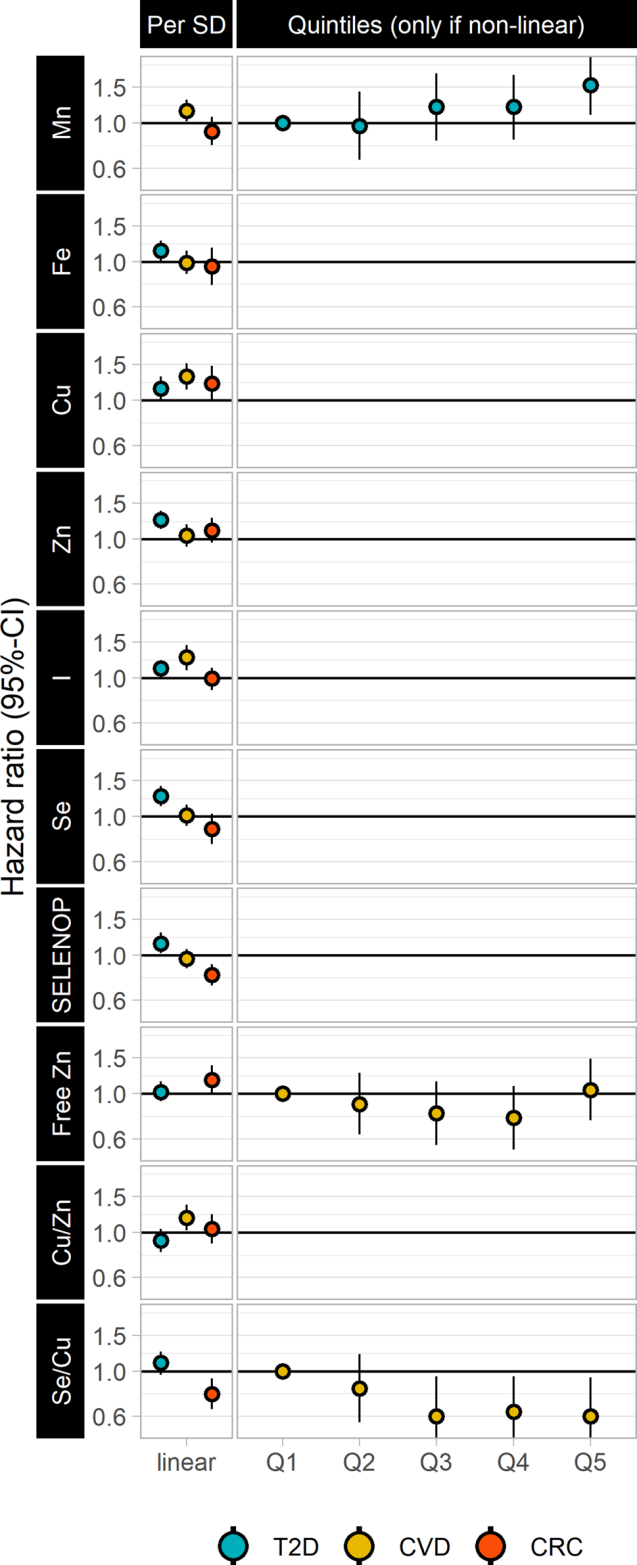
Multivariable-adjusted hazard ratios for T2D, CVD or CRC according to TE concentration, EPIC-Potsdam. Model adjusted for age, sex, educational attainment, BMI, waist circumference, smoking status, physical activity, alcohol intake, vitamin and mineral preparations, prevalent hypertension, anti-hypertensive medication, lipid-lowering medication, and Mediterranean score. *Mn* manganese, *Fe* iron, *Cu* copper, *Zn* zinc, *I* iodine, *Se* selenium, *SELENOP* selenoprotein P, *Free Zn* free zinc, *Cu/Zn* copper-to-zinc ratio, *Se/Cu* selenium-to-copper ratio

After adjustments for age, sex, education and established T2D risk factors, higher concentrations of Mn, Zn, I, Se, and SELENOP were associated with a higher risk of developing T2D (HR Q5 vs Q1, 95% CI 1.54, 1.10–2.17 for Mn; HR per SD, 95% CI 1.24, 1.12–1.38 for Zn; 1.11, 1.03–1.20 for I; 1.26, 1.12–1.41 for Se; 1.14, 1.02–1.29 for SELENOP) (Fig. 2). In the final model, in which further adjustment was made for the respective other main TEs, higher concentrations of Mn, Zn, I and Se did not markedly change in their association with diabetes risk (HR Q5 vs Q1: 1.56, 1.09–2.22 for Mn; HR per SD, 95% CI 1.18, 1.05–1.33 for Zn; 1.09, 1.01–1.17 for I; 1.19, 1.06–1.34 for Se), while SELENOP was largely attenuated (HR per SD, 95% CI 1.04, 0.92–1.18). No association was observed for Fe, Free Zn, Cu-to-Zn ratio and Se-to-Cu ratio (Supplementary Table 1).

Regarding the TE patterns, in the multi-variable adjusted models, both Factor 1 (Mn–Fe–Zn) and Factor 2 (Cu–I–Se) were positively associated with the risk of developing T2D (HR per SD, 95% CI 1.19, 1.09–1.30 for Factor 1; HR Q5 vs Q1, 95% CI 1.76, 1.21–2.56 for Factor 2). We next conducted a stepwise approach adjusting pattern associations one by one for different TEs. Adjustment for Zn attenuated the relationship between the pattern 1 and the risk of T2D (HR per SD, 95% CI 1.11, 1.00–1.24). For factor 2, the adjustment for Zn and Se had the strongest impact on the association between the pattern and T2D (HR Q5 vs Q1, 95% CI 1.47, 0.98–2.18 with adjustment for Zn and 1.02, 0.54–1.94 with adjustment for Se) (Table 3).

**Table 3:** Results of the stepwise approach: hazard ratios (95% CIs) for T2D, CVD or CRC according to TE patterns adding one by one the TE to model 2, EPIC-Potsdam, imputed sample

	No. of cases/non-cases	Model 1	Model 2	Model 2 + Mn	Model 2 + Fe	Model 2 + Cu	Model 2 + Zn	Model 2 + I	Model 2 + Se
Type 2 diabetes									
Factor 1	705/2036	1.09 (1.01–1.17)	1.19 (1.09–1.30)	1.15 (1.04–1.27)	1.19 (1.07–1.33)	1.19 (1.08–1.30)	1.11 (1.00–1.24)	1.21 (1.11–1.32)	1.17 (1.06–1.28)
Factor 2									
Quintile 1	158/415	1 (reference)	1 (reference)	1 (reference)	1 (reference)	1 (reference)	1 (reference)	1 (reference)	1 (reference)
Quintile 2	119/402	0.94 (0.68–1.29)	0.92 (0.63–1.34)	0.94 (0.65–1.37)	0.91 (0.62–1.32)	0.93 (0.63–1.37)	0.86 (0.59–1.26)	0.89 (0.61–1.31)	0.77 (0.51–1.16)
Quintile 3	150/416	1.02 (0.76–1.36)	0.92 (0.65–1.30)	0.98 (0.69–1.39)	0.92 (0.65–1.31)	0.93 (0.65–1.34)	0.85 (0.59–1.21)	0.89 (0.63–1.26)	0.70 (0.46–1.07)
Quintile 4	137/384	1.24 (0.92–1.67)	1.21 (0.85–1.72)	1.27 (0.89–1.81)	1.22 (0.86–1.74)	1.25 (0.84–1.84)	1.08 (0.75–1.56)	1.45 (0.80–1.64)	0.84 (0.51–1.38)
Quintile 5	141/419	1.66 (1.22–2.27)	1.76 (1.21–2.56)	1.88 (1.29–2.75)	1.74 (1.20–2.54)	1.86 (1.16–2.98)	1.47 (0.98–2.18)	1.59 (1.07–2.38)	1.02 (0.54–1.94)
Cardiovascular disease									
Factor 1	414/2050	1.03 (0.93–1.14)	1.04 (0.93–1.16)	0.96 (0.84–1.09)	1.07 (0.95–1.20)	1.02 (0.92–1.14)	1.02 (0.90–1.16)	1.01 (0.90–1.14)	1.03 (0.93–1.15)
Factor 2	414/2050	1.20 (1.05–1.37)	1.17 (1.02–1.35)	1.21 (1.06–1.38)	1.17 (1.02–1.35)	0.95 (0.78–1.17)	1.18 (1.02–1.36)	1.06 (0.90–1.24)	1.42 (1.13–1.78)
Colorectal cancer									
Factor 1	219/2090	1.00 (0.88–1.37)	0.99 (0.86–1.14)	1.03 (0.88–1.20)	1.03 (0.90–1.19)	0.99 (0.86–1.14)	0.92 (0.77–1.10)	0.99 (0.85–1.15)	1.00 (0.88–1.15)
Factor 2	219/2090	1.00 (0.90–1.11)	0.99 (0.89–1.11)	0.98 (0.87–1.11)	0.99 (0.89–1.11)	0.84 (0.67–1.05)	0.97 (0.85–1.10)	1.01 (0.84–1.21)	1.05 (1.96–1.15)

Model 1: adjusted for age and sex, Model 2: adjusted for age, sex, educational attainment, BMI, waist circumference, smoking status, physical activity, alcohol intake, vitamin and mineral preparations, prevalent hypertension, anti-hypertensive medication, lipid-lowering medication, and Mediterranean score

### TEs and risk of cardiovascular disease

In the spline regression analysis, the shape of the observed associations of each TE, the functional markers, Se-to-Cu ratio and both TE patterns with CVD was linear, while the association for the Se-to-Cu ratio deviated from linearity (Supplementary Fig. 3).

After adjustments for age, sex, education and established risk factors, higher concentrations of Mn, Cu, I and Cu-to-Zn ratio were associated with a higher risk of developing CVD (HR per SD, 95% CI 1.15, 1.02–1.30 for Mn; 1.31, 1.13–1.52 for Cu; 1.26, 1.09–1.45 for I; 1.18, 1.02–1.37 for Cu-to-Zn ratio), whereas, Se-to-Cu ratio was inversely associated with CVD incidence (HR Q5 vs Q1, 95% CI 0.60, 0.39–0.93) (Fig. 2). In the final model, in which further adjustment was made for the respective other main TEs (Mn, Fe, Cu, Zn, I and Se), the associations observed for Mn and Cu were slightly attenuated (HR per SD, 95% CI 1.13, 1.00–1.29 for Mn and 1.22, 1.02–1.44 for Cu), while for I the association strongly attenuated (HR per SD, 95% CI 1.17, 0.99–1.38). No significant associations were observed for Fe, Zn, Se and SELENOP (Supplementary Table 2).

With regard to the TE patterns, no significant association was observed for factor 1, while factor 2 presented a positive association with CVD risk after adjustment for age, sex, education and established CVD risk factors (HR per SD, 95% CI 1.17, 1.02–1.35). After conducting a stepwise approach adding one by one the different TEs to the model, the adjustment for Cu and I attenuated the relationship between the pattern and the risk of T2D (HR per SD, 95% CI 0.95, 0.78–1.17 and 1.06, 0.90–1.24, respectively), whereas the adjustment for Se strengthened the magnitude of the association (HR per SD, 95% CI 1.42, 1.13–1.78) (Table 3).

### TEs and risk of colorectal cancer

In the spline regression analysis, the shapes of all observed associations were linear (Supplementary Fig. 4).

After adjustments for age, sex, education and established risk factors, higher concentrations of SELENOP and Se-to-Cu ratio were associated with a lower risk of developing CRC (HR per SD, 95% CI 0.80, 0.71–0.90 and 0.77, 0.65–0.92, respectively), while Free Zn was observed to associate with borderline significance with increased risk of CRC (HR per SD, 95% CI 1.17, 1.00–1.38) (Fig. 2). In the final model, in which further adjustment was made for the respective other main TEs (Mn, Fe, Cu, Zn, I and Se), Free Zn lost statistical significance (HR per SD, 95% CI 1.16, 0.97–1.38) and the association between SELENOP was similar to that of Se, with higher concentrations being associated with a lower risk of CRC (HR per SD, 95% CI 0.81, 0.72–0.93 for SELENOP and 0.82, 0.69–0.98 for Se). Higher Cu concentrations, however, were associated with a higher risk of developing CRC (HR per SD, 95% CI 1.29, 1.05–1.59) and Zn concentrations were associated with borderline significance (HR per SD, 95% CI 1.14, 1.00–1.30). No associations were observed for the other TEs or the Cu-to-Zn ratio (Supplementary Table 3).

No significant associations were observed between the TE patterns and the risk of developing CRC (Table 3).

In the sensitivity analysis, overall the associations were similar to those reported for all endpoints (data not shown).

## Discussion

We found that selected TE concentrations were associated with incidence of all three outcomes (T2D, CVD or CRC). Most of the observed associations were linear and independent of age, sex, education, adiposity, lifestyle factors or the other TEs. We furthermore identified two major TE patterns that might help understanding the interplay of TEs: Mn–Fe–Zn and Cu–I–Se. We observed a positive association between both patterns and the risk of developing T2D**.** The positive association of pattern 1 with T2D is consistent with the associations observed for individual component TEs (Mn and Zn), while the associations of pattern 2 were largely explainable by Se. In contrast, only the Cu–I–Se pattern was associated with the risk of developing CVD and none with CRC. This likely reflects a dilution of association for some individual components by the lack of associations of other TEs or even contrasting associations among component TEs (Cu and Se for CRC).

Mn deficiency is uncommon given the variety of Mn-containing dietary sources. However, excessive and prolonged Mn exposure has been linked to neurological disorders [33, 34], while studies assessing other chronic diseases seem to be missing. Interestingly, our study suggests the existence of a J-shaped association of serum Mn levels and T2D, with high concentrations being associated with increased risk. Mn has furthermore been implicated in cancer prevention given the role of MnSOD in oncogenic activity and metabolic shifts during early tumorigenesis [35]. A role of Mn in age-related diseases is furthermore supported by genetic studies which have linked polymorphisms in MnSOD to various health outcomes, although not consistently [36]. Still, inconsistency across studies might partly be explainable by interaction with environmental factors like dietary Mn or antioxidant intake. For example, the common MnSOD Val16Ala polymorphism modified the association between an antioxidant-rich Mediterranean diet and breast cancer risk [37]. Still, in our study, we found no association between Mn concentrations and CRC risk.

Likewise, we observed an association between higher Zn concentrations and the risk of developing T2D. Zn is implicated in synthesis, storage, and secretion of insulin, as well as being a signaling molecule after insulin secretion [38]. Zn is transported to cells bound to proteins, predominantly albumin, a2-macroglobulin, and transferrin, but only free Zn ions are biologically active [39]. However, Zn concentrations in biological material have not been consistently related to diabetes risk [40, 41]. In two short-term interventions with Zn supplementation, no effect on HOMA-IR was detectable, nor do RCTs suggest effects on lipid biomarkers related to the metabolic syndrome [42]. Two prospective cohort studies on Zn biomarkers do not support a relationship to coronary heart disease [43]—we similarly did not observe an association for CVD, while a borderline association was found for CRC.

Higher Cu concentrations were associated to the risk of developing CVD and CRC. Similar to Zn, the serum concentrations of Cu are strictly regulated by feedback mechanisms that maintain its concentration within certain ranges independent of acute changes in nutritional intake. We found that high Cu-to-Zn ratio was associated with an increased CVD risk. Similarly, high serum Cu-to-Zn ratio was associated with cardiovascular and all-cause mortality among middle-aged men in the Paris Prospective Study 2 [44], and among elderly subjects in the ilSIRENTE Study [45], as well as to CRC in the EPIC cohort study [46]. In addition, a synergistic effect between the pro-oxidative effects of elevated Cu in the presence of low status of Se, the essential component of antioxidant selenoproteins, has been suggested [47]. Accordingly, we observed an inverse association between Se-to-Cu ratio and the risk of developing CVD as well as CRC.

Interestingly, our results indicate opposite associations of Se and T2D versus CRC. The latter was also observed for SELENOP, a functional biomarker of Se status. Prospective studies have generally shown some benefit of higher Se status on the risk of various cancers [2, 3]. However, findings from intervention trials have been mixed [4], which probably emphasizes that supplementation will confer benefit only if intake is inadequate. A recent systematic analysis, including 13 observational studies with more than 30,000 subjects, and 3 RCTs involving more than 20,000 participants, does support a positive association between Se and odds for T2D, but indicates no increased risk upon supplemental Se intake, i.e., divergent results between observational studies and RCTs [48]. Interestingly, we found a positive association of Se with T2D in our study, but a lack of association between SELENOP and T2D after adjustment for other TEs. This finding may indicate that the underlying etiopathogenic mechanisms are more complex than anticipated, and larger and more refined studies are needed for clarifying the association, especially in view of the different subgroups of patients [49]. Regarding Se status and CVD risk, neither Se nor SELENOP showed a significant association, in contrast to the strong associations observed for Se-deficient subjects and CVD in the Malmö Preventive Project published recently [50]. Nevertheless, the Se-to-Cu ratio emerged as a most sensitive parameter for CVD risk, supporting our hypothesis on the importance of TE patterns as highly informative disease-related biomarkers.

We found that high I concentrations were associated with an increased risk of developing T2D, similarly to the E3N-EPIC cohort [51]. High intakes of I may accelerate the development of thyroid disorders, such as hypothyroidism or hyperthyroidism, and have been described to increase the incidence of autoimmune thyroiditis and thyroid cancer risk in China [52]. Again, it will be highly important to conduct future studies on the relation of I with thyroid disease risk in relation to other TEs, especially Fe and Se, and deduce the predictive or diagnostic relevance of composite TE biomarkers [53].

Iron is the most abundant trace metal in the human organism. Although both low and high levels have been associated with health outcomes [54, 55], we did not observe a significant association between total Fe concentrations with any of our endpoints. Nevertheless, we have previously reported from the EPIC-Potsdam study that high serum ferritin levels were associated with higher risk of T2D, while no significant association was observed for soluble transferrin receptor concentrations [56]. This indicates that total serum Fe is a different and not-linearly overlapping marker of Fe status as compared to ferritin concentrations.

Our study was conducted in a well-characterized study population embedded into the EPIC–Potsdam cohort. The novelty of considering multiple essential TEs and TE ratios as composite biomarkers with validated methods, and assessing their relation with three different outcomes that include some of the most common diseases in terms of incidence and mortality worldwide is a major strength of this study. TEs were measured before disease onset, thereby reducing the possibility that biomarker levels changed as a result of the outcome. Furthermore, follow-up proportions exceeded 90%; and all self-reports on incident T2D, CVD and CRC cases were verified through medical records, treating physicians, or death certificates. Nevertheless, the study has some limitations. We relied on a single baseline blood sample and random measurement errors may have attenuated true relations of TEs and endpoints. The samples were stored until time of analysis. Total elemental concentrations are generally very stable, especially in frozen samples and at the pH of serum (⁓ 7.4). Even if interconversion of elemental species occurred, this would not change the total amount of the elements in solution, unless volatile or insoluble species were created, which is unlikely under these storage conditions [57]. Due to missing data, some values were imputed. Nevertheless, in the sensitivity analyses restricted to individuals with available data, the trends in the associations between TE concentrations and different endpoints were comparable to those from the imputed dataset (data not shown). Finally, given the observational nature of this study, causality cannot be proven and we can only speculate on potential pathways that link TE concentrations to chronic disease development.

In conclusion, our study illustrates several strong associations between TEs and T2D, CVD or CRC. Noteworthy, most associations were independent of other TEs. Positive associations were found between Mn, I, Zn and Se with T2D incidence, Mn, Cu and Cu-to-Zn ratio with CVD incidence, as well as Zn and Cu with CRC incidence. Inverse associations were observed for Se and CRC, contrasting the positive association observed for T2D. SELENOP and the Se-to-Cu ratio were inversely associated to CRC, the latter was also observed for CVD. The contrasting associations found for Se might point towards differential disease-related pathways, and the identification of the highly sensitive Se-to-Cu ratio as novel parameter for CVD risk supports the concept of TE patterns as meaningful biomarkers of human disease.

## Supplementary Information

Below is the link to the electronic supplementary material.

Supplementary file1 (DOCX 1149 KB)
