# Supplementary material for: Trace element profile and incidence of type 2 diabetes, cardiovascular disease and colorectal cancer: results from the EPIC-Potsdam cohort study
Source: Eur J Nutr. 2021 Feb 15;60(6):3267–78. doi: 10.1007/s00394-021-02494-3 (PMC8354864; doi:10.1007/s00394-021-02494-3)
Supplement: Supplementary file 1 — Supplementary file1 (DOCX 1149 KB) [file 394_2021_2494_MOESM1_ESM.docx]

**Trace element profile and incidence of type 2 diabetes, cardiovascular disease and colorectal cancer: results from the EPIC-Potsdam cohort study**

1^st^ author: Maria Cabral

**Electronic Supplementary Material**

**Figure Legends**

**Supplemental Figure 1.** Flow diagram depicting study sample derivation, EPIC-Potsdam. Abbreviations: T2D, type 2 diabetes; CVD, cardiovascular disease; CRC, colorectal cancer.

**Supplemental Figure 2.** Multivariable-Adjusted Hazard Ratios for T2D according to TE concentration, EPIC-Potsdam, imputed sample, n=2,741. Minimum *P* for nonlinearity from the five imputations: 0.021 for Mn, 0.643 for Fe, 0.244 for Cu, 0.672 for Zn, 0.612 for I, 0.467 for Se, 0.390 for SELENOP, 0.318 for Free Zn, 0.071 for Cu/Zn, 0.131 for Se/Cu, 0.233 for Factor 1 and 0.034 for Factor 2. Models were adjusted for age, sex, educational attainment, BMI, waist circumference, smoking status, physical activity, alcohol intake, vitamin and mineral preparations, prevalent hypertension, anti-hypertensive medication, lipid-lowering medication and Mediterranean score. Model for Mn, Fe, Cu, Zn, I Se, SELENOP and Free Zn were further adjusted for other TE (each TE was further adjusted for the respective other TE included in the PCA: Mn, Fe, Cu, Zn, I and Se). TE were log-transformed and Z-standardized (mean=0, SD=1).

**Supplemental Figure 3.** Multivariable-Adjusted Hazard Ratios for CVD according to TE concentration, EPIC-Potsdam, imputed sample, n=2,464. Minimum *P* for nonlinearity from the five imputations: 0.482 for Mn, 0.612 for Fe, 0.776 for Cu, 0.086 for Zn, 0.351 for I, 0.747 for Se, 0.501 for SELENOP, 0.018 for Free Zn, 0.353 for Cu/Zn, 0.003 for Se/Cu, 0.573 for Factor 1 and 0.323 for Factor 2. Models were adjusted for age, sex, educational attainment, BMI, waist circumference, smoking status, physical activity, alcohol intake, vitamin and mineral preparations, prevalent hypertension, anti-hypertensive medication, lipid-lowering medication and Mediterranean score. Model for Mn, Fe, Cu, Zn, I Se, SELENOP and Free Zn were further adjusted for other TE (each TE was further adjusted for the respective other TE included in the PCA: Mn, Fe, Cu, Zn, I and Se). TE were log-transformed and Z-standardized (mean=0, SD=1).

**Supplemental** **Figure 4.** Multivariable-Adjusted Hazard Ratios for CRC according to TE concentration, EPIC-Potsdam, imputed sample, n=2,309. Minimum *P* for nonlinearity from the five imputations: 0.465 for Mn, 0.288 for Fe, 0.370 for Cu, 0.172 for Zn, 0.274 for I, 0.299 for Se, 0.609 for SELENOP, 0.734 for Free Zn, 0.350 for Cu/Zn, 0.231 for Se/Cu, 0.541 for Factor 1 and 0.645 for Factor 2. Models were adjusted for age, sex, educational attainment, BMI, waist circumference, smoking status, physical activity, alcohol intake, vitamin and mineral preparations, prevalent hypertension, anti-hypertensive medication, lipid-lowering medication and Mediterranean score. Model for Mn, Fe, Cu, Zn, I Se, SELENOP and Free Zn were further adjusted for other TE (each TE was further adjusted for the respective other TE included in the PCA: Mn, Fe, Cu, Zn, I and Se). TE were log-transformed and Z-standardized (mean=0, SD=1).

**Supplemental Figure 1**

**
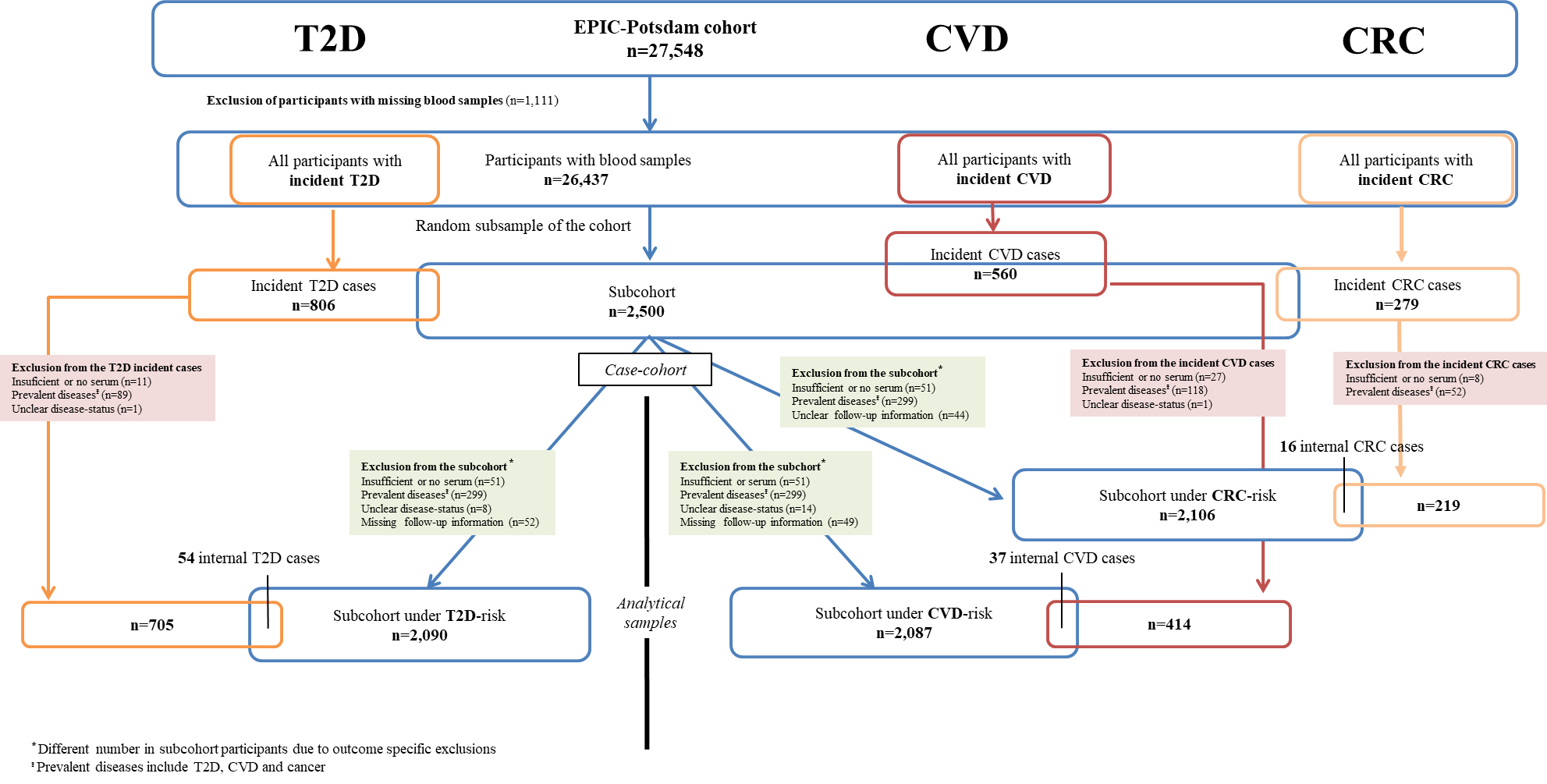
**

**Supplemental Figure 2**

| 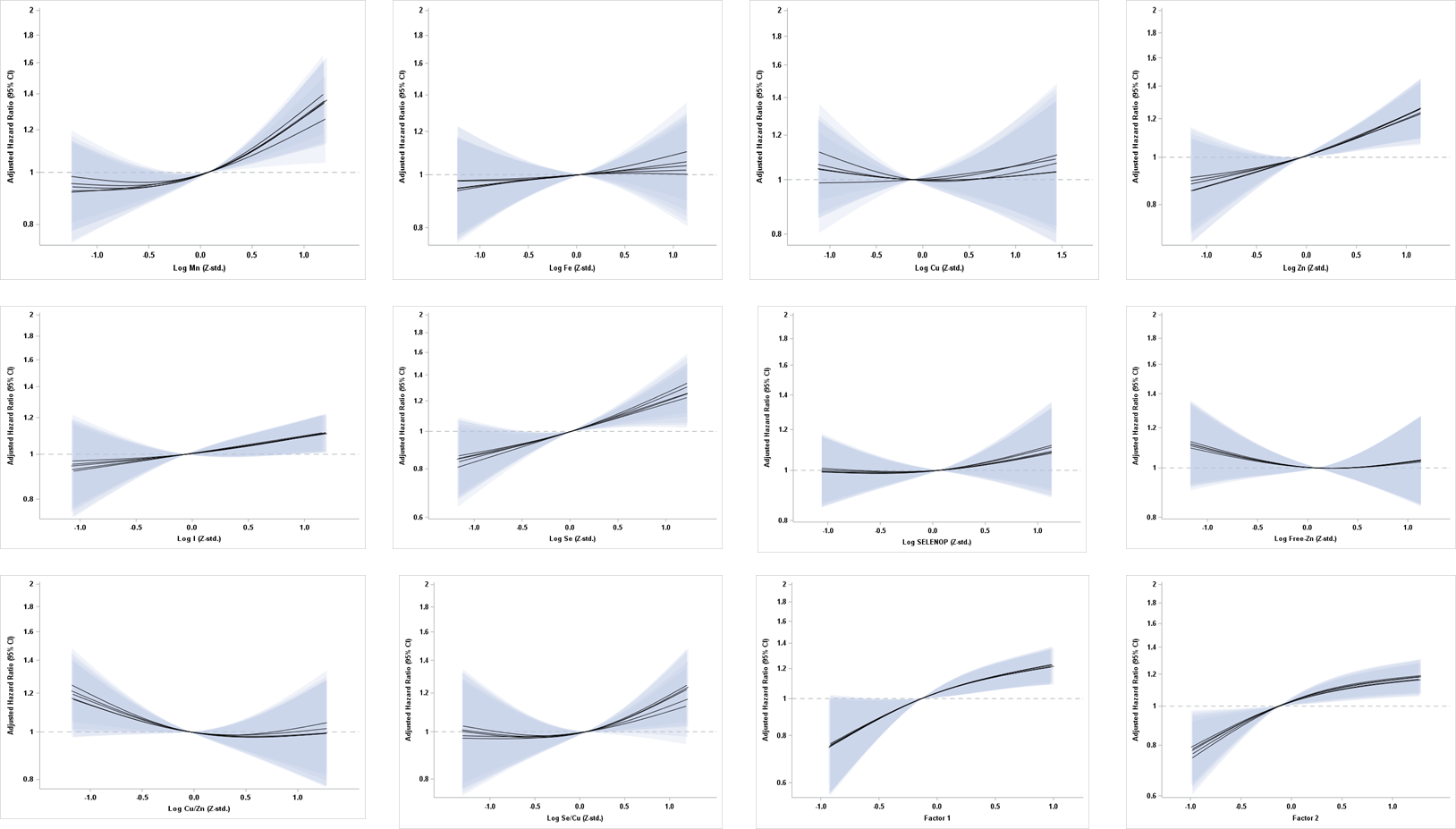 |
| --- |

**Supplemental Figure 3**

| 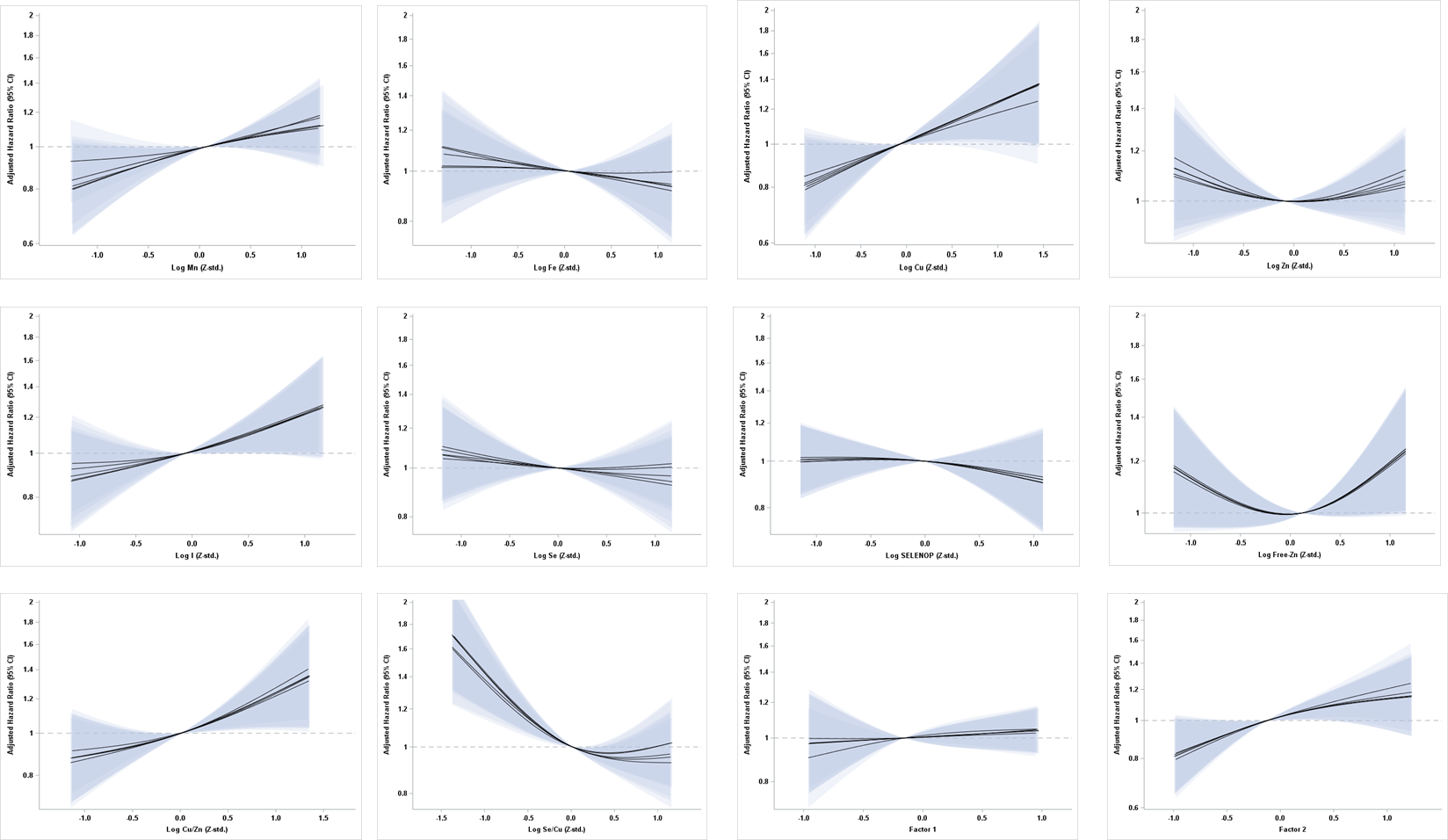 |
| --- |

**Supplemental Figure 4**

| 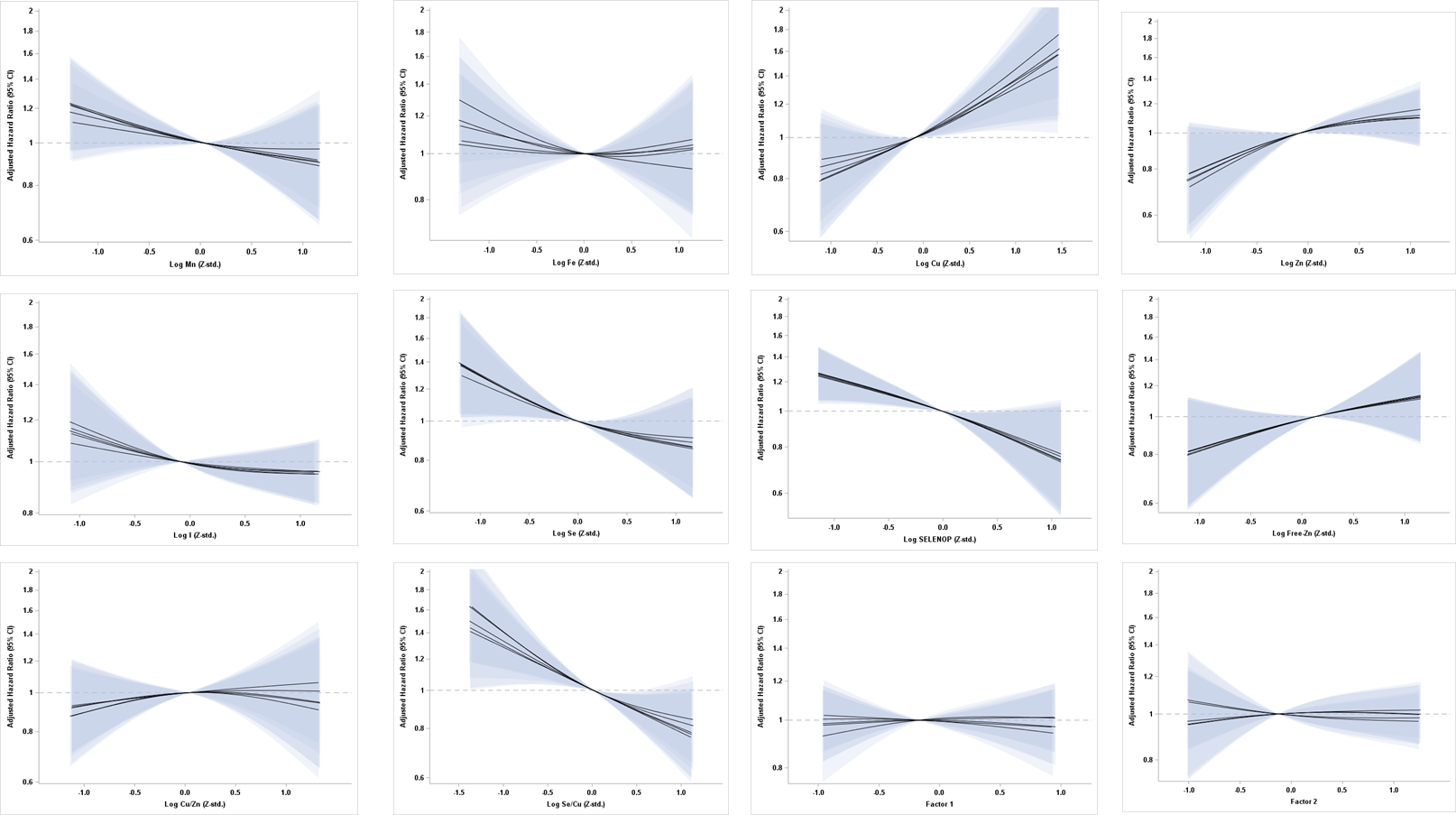 |
| --- |

**Supplemental table 1. Hazard ratios (95% CIs) for T2D according to TE concentrations, EPIC-Potsdam, imputed sample, n=2,741.**

|  | **Type 2 Diabetes** | | | |
| --- | --- | --- | --- | --- |
|  | **No. of cases/**  **non-cases** | **Hazard ratio**  **(95% CI),^1^** | **Hazard ratio**  **(95% CI),^2^** | **Hazard ratio**  **(95% CI),^3^** |
| **Manganese** |  |  |  |  |
| **Quintile 1** | 115/381 | 1 (reference) | 1 (reference) | 1 (reference) |
| **Quintile 2** | 141/435 | 1.06 (0.78-1.44) | 0.97 (0.66-1.43) | 1.03 (0.69-1.52) |
| **Quintile 3** | 143/409 | 1.16 (0.85-1.59) | 1.20 (0.82-1.75) | 1.23 (0.83-1.81) |
| **Quintile 4** | 131/407 | 1.20 (0.89-1.63) | 1.20 (0.83-1.72) | 1.29 (0.89-1.87) |
| **Quintile 5** | 175/404 | 1.50 (1.12-2.00) | 1.54 (1.10-2.17) | 1.56 (1.09-2.22) |
| **Iron** | 705/2036 | 1.05 (0.96-1.15) | 1.13 (1.00-1.27) | 1.04 (0.92-1.17) |
| **Copper** | 705/2036 | 1.22 (1.09-1.36) | 1.14 (0.99-1.31) | 1.00 (0.86-1.17) |
| **Zinc** | 705/2036 | 1.19 (1.10-1.30) | 1.24 (1.12-1.38) | 1.18 (1.05-1.33) |
| **Iodine** | 705/2036 | 1.12 (1.04-1.20) | 1.11 (1.03-1.20) | 1.09 (1.01-1.17) |
| **Selenium** | 705/2036 | 1.16 (1.05-1.27) | 1.26 (1.12-1.41) | 1.19 (1.06-1.34) |
| **Selenoprotein P** | 705/2036 | 1.19 (1.07-1.31) | 1.14 (1.02-1.29) | 1.04 (0.92-1.18) |
| **Free zinc** | 705/2036 | 0.92 (0.84-1.02) | 1.02 (0.92-1.15) | 0.96 (0.85-1.08) |
| **Cu-to-Zn ratio** | 705/2036 | 0.97 (0.88-1.08) | 0.91 (0.80-1.04) | - |
| **Se-to-Cu ratio** | 705/2036 | 0.97 (0.87-1.07) | 1.10 (0.96-1.25) | - |

^ᵻ^TE (continuous) were log-transformed and Z-standardized (mean=0, SD=1)

Model 1. Adjusted for age and sex, Model 2. Adjusted for age, sex, educational attainment, BMI, waist circumference, smoking status, physical activity, alcohol intake, vitamin and mineral preparations, prevalent hypertension, anti-hypertensive medication, lipid-lowering medication, and Mediterranean score, Model 3. Model 2 plus other TE (each TE was further adjusted for the respective other TE included in the PCA: Mn, Fe, Cu, Zn, I and Se).

**Supplemental table 2. Hazard ratios (95% CIs) for CVD according to TE concentrations, EPIC-Potsdam, imputed sample, n=2,464.**

|  | **Cardiovascular Disease** | | | |
| --- | --- | --- | --- | --- |
|  | **No. of cases/**  **non-cases** | **Hazard ratio**  **(95% CI),^1^** | **Hazard ratio**  **(95% CI),^2^** | **Hazard ratio**  **(95% CI),^3^** |
| **Manganese** | 414/2050 | 1.12 (1.00-1.26) | 1.15 (1.02-1.30) | 1.13 (1.00-1.29) |
| **Iron** | 414/2050 | 1.00 (0.88-1.14) | 0.99 (0.87-1.13) | 0.95 (0.83-1.09) |
| **Copper** | 414/2050 | 1.38 (1.20-1.59) | 1.31 (1.13-1.52) | 1.22 (1.02-1.44) |
| **Zinc** | 414/2050 | 1.05 (0.93-1.17) | 1.04 (0.91-1.18) | 1.00 (0.88-1.15) |
| **Iodine** | 414/2050 | 1.34 (1.17-1.54) | 1.26 (1.09-1.45) | 1.17 (0.99-1.38) |
| **Selenium** | 414/2050 | 1.00 (0.88-1.12) | 1.01 (0.90-1.14) | 0.96 (0.84-1.10) |
| **Selenoprotein P** | 414/2050 | 0.97 (0.86-1.08) | 0.96 (0.86-1.07) | 0.97 (0.85-1.10) |
| **Free zinc** |  |  |  |  |
| **Quintile 1** | 100/391 | 1 (reference) | 1 (reference) | 1 (reference) |
| **Quintile 2** | 83/419 | 0.86 (0.61-1.20) | 0.89 (0.63-1.27) | 0.94 (0.65-1.35) |
| **Quintile 3** | 74/405 | 0.75 (0.53-1.06) | 0.80 (0.56-1.15) | 0.85 (0.59-1.24) |
| **Quintile 4** | 74/419 | 0.73 (0.52-1.04) | 0.76 (0.53-1.09) | 0.80 (0.55-1.16) |
| **Quintile 5** | 83/416 | 0.92 (0.65-1.30) | 1.04 (0.74-1.49) | 1.11 (0.76-1.63) |
| **Cu-to-Zn ratio** | 414/2050 | 1.22 (1.06-1.40) | 1.18 (1.02-1.37) | - |
| **Se-to-Cu ratio** |  |  |  |  |
| **Quintile 1** | 92/415 | 1 (reference) | 1 (reference) | - |
| **Quintile 2** | 79/405 | 0.71 (0.49-1.03) | 0.82 (0.56-1.21) | - |
| **Quintile 3** | 77/407 | 0.51 (0.33-0.79) | 0.60 (0.38-0.94) | - |
| **Quintile 4** | 78/418 | 0.50 (0.34-0.73) | 0.63 (0.42-0.94) | - |
| **Quintile 5** | 88/405 | 0.49 (0.33-0.74) | 0.60 (0.39-0.93) | - |

^ᵻ^TE (continuous) were log-transformed and Z-standardized (mean=0, SD=1).

Model 1. Adjusted for age and sex, Model 2. Adjusted for age, sex, educational attainment, BMI, waist circumference, smoking status, physical activity, alcohol intake, vitamin and mineral preparations, prevalent hypertension, anti-hypertensive medication, lipid-lowering medication, and Mediterranean score, Model 3. Model 2 plus other TE (each TE was further adjusted for the respective other TE included in the PCA: Mn, Fe, Cu, Zn, I and Se).

**Supplemental table 3. Hazard ratios (95% CIs) for CRC according to TE concentrations, EPIC-Potsdam, imputed sample, n=2,309.**

|  | **Colorectal Cancer** | | | |
| --- | --- | --- | --- | --- |
|  | **No. of cases/**  **non-cases** | **Hazard ratio**  **(95% CI),^1^** | **Hazard ratio**  **(95% CI),^2^** | **Hazard ratio**  **(95% CI),^3^** |
| **Manganese** | 219/2090 | 0.94 (0.81-1.09) | 0.91 (0.78-1.07) | 0.89 (0.76-1.05) |
| **Iron** | 219/2090 | 0.96 (0.79-1.16) | 0.95 (0.77-1.17) | 0.95 (0.77-1.17) |
| **Copper** | 219/2090 | 1.22 (1,01-1.47) | 1.21 (0.99-1.48) | 1.29 (1.05-1.59) |
| **Zinc** | 219/2090 | 1.09 (0.97-1.24) | 1.10 (0.96-1.27) | 1.14 (1.00-1.30) |
| **Iodine** | 219/2090 | 0.96 (0.84-1.11) | 0.99 (0.87-1.12) | 0.94 (0.82-1.09) |
| **Selenium** | 219/2090 | 0.88 (0.75-1.04) | 0.87 (0.73-1.03) | 0.82 (0.69-0.98) |
| **Selenoprotein P** | 219/2090 | 0.80 (0.72-0.89) | 0.80 (0.71-0.90) | 0.81 (0.72-0.93) |
| **Free zinc** | 219/2090 | 1.17 (1.00-1.37) | 1.17 (1.00-1.38) | 1.16 (0.97-1.38) |
| **Cu-to-Zn ratio** | 219/2090 | 1.05 (0.90-1.23) | 1.04 (0.88-1.23) | - |
| **Se-to-Cu ratio** | 219/2090 | 0.77 (0.66-0.91) | 0.77 (0.65-0.92) | - |

^ᵻ^TE (continuous) were log-transformed and Z-standardized (mean=0, SD=1).

Model 1. Adjusted for age and sex, Model 2. Adjusted for age, sex, educational attainment, BMI, waist circumference, smoking status, physical activity, alcohol intake, vitamin and mineral preparations, prevalent hypertension, anti-hypertensive medication, lipid-lowering medication, and Mediterranean score, Model 3. Model 2 plus other TE (each TE was further adjusted for the respective other TE included in the PCA: Mn, Fe, Cu, Zn, I and Se).
